# What drives water conservation in the supply chain of the Yellow River Basin? An empirical analysis based on SPD

**DOI:** 10.1371/journal.pone.0306519

**Published:** 2024-08-01

**Authors:** Yanhua Shi, Shanshan Fan, Qianqian Xiao, Ziyu Li

**Affiliations:** 1 School of Economics and Finance, Hohai University, Changzhou, China; 2 Business School, Hohai University, Changzhou, China; Chengdu University of Information Technology, CHINA

## Abstract

Industrial water saving is an objective requirement for the high-quality development of the Yellow River Basin, as water resource is the largest rigid constraint. In this study, water resources input-output model, structural decomposition analysis (SDA) and structural path analysis (SPA) were constructed to decompose the driving factors of total water use in typical water-deficient provinces (Ningxia, Shanxi, and Henan) in China’s Yellow River Basin, to calculate their water use at each production stage and identify their key water-saving pathways. The results were as follows: (i) Water intensity had the most obvious impact on total water saving, resulting in efficiency improvements of 81.39%, 9.21%, and 78.45% for each province, respectively. The next factor was the final demand structure, which suppressed total water-saving efforts by 24.23%, 11.52%, and 113.12% in the respective provinces. (ii) The key water-saving paths in the typical water-deficient provinces of the Yellow River Basin were primarily centered around Sector 1. (iii) Water intensity had a strong water-saving effect on the key paths in the three provinces, with contribution rates of 100.42%, 59.02%, and 42.34% for Ningxia, Henan, and Shanxi, respectively. Final demand also contributed to water-saving in the key paths of Shanxi and Henan, with contribution rates of 35.06% and 28.23%, respectively. However, it inhibited water-saving efforts in the key paths of Ningxia, reducing it by 8.64%. Policy measures should be tailored to local conditions.

## Introduction

Water resources are the foundation of human survival and development and play a vital role in the sustainable development of society [1]. Water resources influence a society’s development and prospects more than any other resource [2]. As one of the important sources of freshwater supply [3], rivers also face water scarcity problems, particularly in the Yellow River basin [4]. Being a typical resource-based water-deficient region, the per capita water resources in the Yellow River basin amount to 408 m^3^ [5], approximately 20% of the national average and far below the internationally recognized “extreme water scarcity standard” of 500 m^3^ per capita. Water scarcity has become a major constraint to the high-quality development of the Yellow River Basin [6]. The Yellow River Basin is divided into three regions: upstream, midstream and downstream, which show completely different characteristics in terms of water resources and economic structure [7]. In 2021, Qinghai Province in the upstream region had a per capita water resource volume of 14,190 m^3^ [5], while Ningxia Hui Autonomous Region only had 128 m^3^ per capita [5]. Apart from Qinghai, Sichuan, and Inner Mongolia, all other upstream provinces have per capita water resource levels below the national average. In addition, the five provinces in the middle and lower reaches of the Yellow River Basin have a high overall development scale and quality of traditional industries, with Shanxi Province standing out due to its developed energy production [8]. In 2020, water use per 10,000 yuan of GDP in Shanxi Province was 40.82 m^3^, the highest among the middle reaches, indicating lower water utilization efficiency in Shanxi Province. Henan Province, located in the downstream region, is a major agricultural province consistently leading in the primary industry-added value and grain production nationwide [9]. However, agriculture is often regarded as the largest water user but with the lowest value [10]. Given the significant economic and resource characteristics across the Yellow River basin’s upstream, middle, and downstream regions, it is necessary to select representative provinces for precise research on water resource management issues [11].

For a long time, water resources management has often been limited to the perspective of direct water use [12–14], ignoring the indirect water use associated with commodity circulation. Allan first proposed the concept of virtual water, which refers to the amount of water resources consumed in service or material formation [15]. Combining the economic and social system with the water resources system can more accurately grasp the depth law of water resources utilization in regional economic and social development, and provide a new perspective for water resources management. Therefore, the potential for water saving from the perspective of virtual water will be actively explored in this study.

Currently, quantitative studies on virtual water can be mainly categorized into two methods: the bottom-up approach, represented by the production tree method [16, 17], and the top-down approach, represented by the input-output method [18, 19]. The former overlooks the comprehensiveness of industry products and the interconnection of water usage among industries [20–22]. On the other hand, the latter can trace both direct and indirect water consumption along the entire supply chain [23], describing the total water consumption of the final demand product within the industrial chain and better reflecting inter-industry water usage relationships. Zhang et al., Wang et al., and Wu et al. have introduced the water resources input-output model to calculate direct and complete water coefficients at the national, regional, and sector levels, respectively, and analyze the pattern of virtual water flows [23–25]. However, the water resources input-output model only provides a static analytical framework for virtual water. It is necessary to further analyze the drivers and impacts of industrial water usage at a deeper level, investigate the underlying factors behind virtual water flow, and implement more effective water-saving measures.

The Index Decomposition Analysis (IDA) and Structural Decomposition Analysis (SDA) [26–28] are adopted in mainstream research to reveal the contribution and interplay of various factors to the overall index changes. IDA allows for the quantification of key driving factors that influence water consumption [29, 30]. However, it does not consider factors closely associated with water use variations, such as technological advancements, efficiency changes, and substitution among factors, resulting in significant limitations in its explanatory power. On the other hand, SDA is a method based on input-output models that analyzes the direct and indirect impacts of certain driving factors on a specific index, offering a deeper understanding of the sources of water changes [31–33] and extending the depth of decomposition analysis. For instance, Huang et al. [33] utilized the SDA method to quantitatively decompose the influencing factors based on the gains and losses of virtual water trade and found that the growth in import and export volumes was the primary factor driving the increase in virtual water trade, highlighting the need for corresponding strategies in virtual water trade.

Based on the application scenarios described earlier for the water resources input-output method, it is evident that water resources input-output analysis can reflect the overall flow of water resources among sectors but fails to differentiate the specific production levels where water consumption occurs. In other words, water resources input-output analysis alone cannot fully capture the water transmission chain. It is also unable to trace the water flows between supply chain nodes and identify the focal sectors that play a crucial role in these water flows. Consequently, traditional water input-output analyses can only provide vague policy recommendations, such as adjusting industrial structures, without offering targeted water-saving policies at each production stage, distribution, circulation, and consumption. To address this, it is imperative to understand the water usage of each sector at various stages and depict a complete water transmission pathway that aligns with the industrial chain. This is where the Structural Path Analysis (SPA) serves as a key tool [34, 35]. For instance, Pang et al. [34], used environmental extended multi-regional input-output analysis, quantified the water footprints associated with the construction industry across different countries. They then employed SPA to decompose these environmental footprints into more refined flow paths and determine the critical junctures at the intersection of paths.

However, neither the factor decomposition of direct water consumption nor the structural path analysis of industrial water usage can further reflect the varying influence of the same factor on water consumption at different hierarchical economic activities. Therefore, Wood et al. [36], combining the SDA and SPA models, proposed the Structural Path Decomposition (SPD) model to conduct comparative analyses of resource (or environmental) factors from an industry chain perspective. After clarifying the specific industry pathways of resource flows (or environmental changes), they proceeded to unravel the impacts of different factors on each pathway. Zhang et al. [37, 38], combining SDA and SPA, identified the primary driving factors and pathways of carbon emissions growth in China. However, existing research has not yet combined SDA and SPA in the field of virtual water. Hence, in this study, SPD will be utilized to investigate industrial water, which not only allows for the decomposition of driving factors but also facilitates a further breakdown of the influences of various factors at different production stages and production chains. This will enable a deeper observation and the ability to implement targeted strategies.

This study encompasses several aspects: Firstly, a water input-output model is established. Building upon this, the SDA method is used to decompose the impacts of water intensity effects, input-output structural effects (or direct consumption coefficient effects), and final demand effects on changes in water usage at various levels of the production system. Then, employing the SPA method, the water consumption and water-saving volume of each production stage and production chain are calculated, enabling us to track the production chains with the most significant water-saving effects and analyze their characteristics. Finally, the SPD method is adopted to identify the key driving factors contributing to water consumption changes along key water-saving paths.

This study brings forth innovative aspects and contributions. Firstly, it advances the research scope by focusing on a novel research subject. Specifically, we select representative water-stressed provinces within the Yellow River Basin to precisely analyze their water issues, aiming to achieve differentiated governance. Scholars such as Zhang et al. [39] have pointed out that previous studies mostly operated at coarse spatial resolutions, dividing the entire basin into upstream, midstream, and downstream units or examining the entire basin, providing limited quantitative insights into spatial variations within the basin. In contrast, a provincial-scale analysis is delved into in the research, overcoming the limitations of macro-level investigations of the Yellow River Basin. Secondly, it introduces methodological innovation. In previous studies, the SPD method has been widely employed to investigate factors affecting carbon emissions. For instance, Gui et al. [40] utilized SDA and SPD to analyze the key factors and supply chain pathways driving changes in carbon emissions in China. The SPD model demonstrates excellent performance and portability. Some scholars [41] have also applied the SPD method to investigate the key path changes that drive four major categories of resources (biomass, metals, non-metals, and fossil fuels) consumption and the key factors that lead to consumption changes in each path. In this study, the SPD model is creatively applied to the field of virtual water, combining SDA and SPA. The results of this paper provide important decision-making references for water conservation at source, industrial linkage, and key sectors. Thirdly, this study innovatively categorizes water usage into different levels (total water consumption, intermediate production stage water usage, and industrial chain water usage) to thoroughly investigate its inherent influencing factors, enriching the research framework on virtual water and facilitating systematic water conservation efforts.

## Materials and methods

### Water resources input-output model

Water resources input-output model is based on input-output tables. An input-output table is a checkerboard table that reflects the sources of inputs and the destination of outputs for each industry [42]. In an input-output table, the horizontal data represent the distribution of each industry’s output throughout the economic system; the vertical data represent the intermediate inputs from all industries that each industry requires to produce its output. Table 1 shows a sample input-output table.

**Table 1:** Sample input-output table.

Allocation destination/Input source		Intermediate use				Final use	Total output
		Sector 1	Sector 2	……	Sector n		
Intermediate input	Sector 1	x_11_	x_12_	……	x_1n_	Y_1_	X_1_
	Sector 2	x_21_	x_22_	……	x_2n_	Y_2_	X_2_
	……	……	……	……	……	……	……
	Sector n	x_n1_	x_n2_	……	x_nn_	Y_n_	X_n_
Total input		X_1_	X_2_	……	X_n_		

The horizontal relationship equation of an input-output table can be expressed as Intermediate input + Final demand = Total output. It can be represented by the formula as Eq (1):

$$\sum_{j=1}^{n}x_{ij}+Y_{i}=X_{i}\;(i=1,2,\cdots ,n)$$ (1)

x_ij_ represents the quantity of products produced by industry i allocated to industry j. Y_i_ represents the final demand for industry i, including household consumption, government consumption, investment, exports, etc. X_i_ represents the total output of industry i.

a_ij_ represents the direct consumption coefficient, which means the quantity of products from industry i consumed in per unit of product produced by industry j. The calculation formula for the direct consumption coefficient is shown in Formula (2):

$$a_{ij}=\frac{x_{ij}}{X}\;(i,j=1,2,\cdots ,n)$$ (2)

By substituting Eq (2) into Eq (1), we can obtain Eq (3):

$$\sum_{j=1}^{n}a_{ij}X_{j}+Y_{i}=X_{i}\;(i=1,2,\cdots ,n)$$ (3)

Writing Eq (3) in matrix form, we get Eq (4):

AX+Y=X (4)

Eq (4) can also be written in the form of Eq (5). (*I*−*A*)^−1^ is the Leontief inverse matrix, so let it be L.


$$X={\left(I-A\right)}^{-1}Y$$ (5)

A water resources input-output model can be established by incorporating water-related information into the input-output model [43]. The total water consumption matrix W is equal to the water intensity matrix multiplied by the total output matrix, expressed as Formula (6):

W=RX=RLY (6)

The matrix Y can also be replaced with Eq (7). In Eq (7), Y_str_ represents the matrix of the sector structure of final demand, where the matrix elements represent the proportion of the volume of a specific type of final demand for one industry to the scale of a specific type of final demand. ${\hat{Y}}_{cat}$ is the diagonal matrix of the type structure of final demand, where the matrix elements represent the proportion of the scale of a specific type of final demand to the total final demand. Y_T_ is the total final demand.


$$Y=Y_{str}{\hat{Y}}_{cat}Y_{T}$$ (7)

By substituting Eq (7) into Formula (6), we obtain Formula (8):

$$W=RLY_{str}{\hat{Y}}_{cat}Y_{T}$$ (8)

### Structural decomposition analysis

Structural decomposition analysis (SDA) is a method based on input-output model to analyze certain factors’ direct and indirect influence on an index [44]. The derivation process of SDA is as follows.

Firstly, based on Formula (8), the variation in water consumption (*ΔW*) can be expressed as Formula (9). Subscripts 0 and 1 indicate the beginning and the end years, respectively.


$$\begin{array}{l} \Delta W=R_{1}L_{1}Y_{str,1}{\hat{Y}}_{cat,1}Y_{T,1}-R_{0}L_{0}Y_{str,0}{\hat{Y}}_{cat,0}Y_{T,0}\\ \;=(R_{1}-R_{0})L_{0}Y_{str,0}{\hat{Y}}_{cat,0}Y_{T,0}+R_{1}(L_{1}-L_{0})Y_{str,0}{\hat{Y}}_{cat,0}Y_{T,0}\\ \;+R_{1}L_{1}(Y_{str,1}-Y_{str,0}){\hat{Y}}_{cat,0}Y_{T,0}+R_{1}L_{1}Y_{str,1}({\hat{Y}}_{cat,1}-{\hat{Y}}_{cat,0})Y_{T,0}\\ \;+R_{1}L_{1}Y_{str,1}{\hat{Y}}_{cat,1}(Y_{T,1}-Y_{T,0})\\ \;=\Delta RL_{0}Y_{str,0}{\hat{Y}}_{cat,0}Y_{T,0}+R_{1}\Delta LY_{str,0}{\hat{Y}}_{cat,0}Y_{T,0}\\ \;+R_{1}L_{1}\Delta Y_{str}{\hat{Y}}_{cat,0}Y_{T,0}+R_{1}L_{1}Y_{str,1}\Delta {\hat{Y}}_{cat}Y_{T,0}\\ \;+R_{1}L_{1}Y_{str,1}{\hat{Y}}_{cat,1}\Delta Y_{T} \end{array}$$ (9)

Secondly, the variation in water consumption (*ΔW*) can also be expressed as Formula (10):

$$\begin{array}{l} \Delta W=R_{1}L_{1}Y_{str,1}{\hat{Y}}_{cat,1}Y_{T,1}-R_{0}L_{0}Y_{str,0}{\hat{Y}}_{cat,0}Y_{T,0}\\ \;=(R_{1}-R_{0})L_{1}Y_{str,1}{\hat{Y}}_{cat,1}Y_{T,1}+R_{0}(L_{1}-L_{0})Y_{str,1}{\hat{Y}}_{cat,1}Y_{T,1}\\ \;+R_{0}L_{0}(Y_{str,1}-Y_{str,0}){\hat{Y}}_{cat,1}Y_{T,1}+R_{0}L_{0}Y_{str,0}({\hat{Y}}_{cat,1}-{\hat{Y}}_{cat,0})Y_{T,1}\\ \;+R_{0}L_{0}Y_{str,0}{\hat{Y}}_{\mathrm{cat},0}(Y_{T,1}-Y_{T,0})\\ \;=\Delta RL_{1}Y_{str,1}{\hat{Y}}_{cat,1}Y_{T,1}+R_{0}\Delta LY_{str,1}{\hat{Y}}_{cat,1}Y_{T,1}\\ \;+R_{0}L_{0}\Delta Y_{str}{\hat{Y}}_{cat,1}Y_{T,1}+R_{0}L_{0}Y_{str,0}\Delta {\hat{Y}}_{cat}Y_{T,1}\\ \;+R_{0}L_{0}Y_{str,0}{\hat{Y}}_{cat,0}\Delta Y_{T} \end{array}$$ (10)

From Formula (9) and (10), Formula (11) can be derived:

$$\begin{array}{l} \Delta W=\frac{1}{2}(\Delta RL_{0}Y_{str,0}{\hat{Y}}_{cat,0}Y_{T,0}+\Delta RL_{1}Y_{str,1}{\hat{Y}}_{cat,1}Y_{T,1})\\ \;+\frac{1}{2}(R_{1}\Delta LY_{str,0}{\hat{Y}}_{cat,0}Y_{T,0}+R_{0}\Delta LY_{str,1}{\hat{Y}}_{cat,1}Y_{T,1})\\ \;+\frac{1}{2}(R_{1}L_{1}\Delta Y_{str}{\hat{Y}}_{cat,0}Y_{T,0}+R_{0}L_{0}\Delta Y_{str}{\hat{Y}}_{cat,1}Y_{T,1})\\ \;+\frac{1}{2}(R_{1}L_{1}Y_{str,1}\Delta {\hat{Y}}_{cat}Y_{T,0}+R_{0}L_{0}Y_{str,0}\Delta {\hat{Y}}_{cat}Y_{T,1})\\ \;+\frac{1}{2}(R_{1}L_{1}Y_{str,1}{\hat{Y}}_{cat,1}\Delta Y_{T}+R_{0}L_{0}Y_{str,0}{\hat{Y}}_{cat,0}\Delta Y_{T}) \end{array}$$ (11)

The right-hand side of Formula (11) consists of five parts. The first row represents the change in water consumption caused by variations in water intensity. The second row represents the change in water consumption caused by variations in input-output structure. The third row represents the change in water consumption caused by variations in the structure of final demand. The fourth row represents the change in water consumption caused by variations in the type of final demand. The fifth row represents the change in water consumption caused by variations in the total scale of final demand. In the “Analysis of water consumption at macro aggregate level” section in this paper, the SDA method is used to decompose the total water consumption changes in the three provinces of the Yellow River Basin from 2012 to 2017 into five driving factors: water intensity, input-output structure, final demand structure, final demand type, and final demand scale.

### Structural path analysis

Structural path analysis (SPA) was first put forward by Defourny in 1984 [45]. This method can decompose water consumption into infinite paths in the production system; each pathway represents the process of water resources transferring with the intermediate input of products. The derivation process of SDA is as follows.

Firstly, the Leontief inverse matrix is expanded by power series approximation [46], and Eq (12) can be derived:

$$L=(I-A)-1=I+A+A^{2}+A^{3}+\cdots$$ (12)

Secondly, by substituting Eq (12) into Formula (6) [47], we obtain Formula (13):

$$W=RLY=RIY+RAY+RA^{2}Y+RA^{3}Y+\cdots$$ (13)

In Formula (13), RIY is the direct water consumption in the production process of products or services consumed by residents. RA^n^Y represents the water consumption used by the N-level sector for producing products after an increase in demand from higher-level sectors [48]. The higher the number of levels, the less water consumption, so it is necessary to cut off the production chain appropriately [49]. When the water consumption ratio of a certain level is lower than the specified threshold, the remaining nodes are excluded. According to the calculation results, this paper selects 1% as the threshold [48]. In the “Analysis of water consumption during the intermediate production stages” section of this paper, SPA method is used to calculate the water consumption in the production stages of three provinces in 2012 and 2017. RIY represents the water consumption in phase 0, RAY represents the water consumption in phase 1, RA^2^Y represents the water consumption in phase 2, RA^3^Y represents the water consumption in phase 3, and so on for phases 4, 5 to +∞.

Thirdly, specific to the elements of each matrix in Formula (13), Formula (13) can be further expressed as Formula (14) [50]:

$$\begin{array}{l} W=\sum_{i=1}^{n}r_{i}y_{i}+\sum_{i=1}^{n}\sum_{j=1}^{n}r_{i}a_{ij}y_{j}+\sum_{i=1}^{n}\sum_{k=1}^{n}\sum_{j=1}^{n}r_{i}a_{ik}a_{kj}y_{j}+\cdots \\ \;=\sum_{i=1}^{n}r_{i}(y_{R,i}+y_{U,i}+y_{G,i}+y_{F,i}+y_{I,i}+y_{E,i}+y_{P,i})\\ \;+\sum_{i=1}^{n}\sum_{j=1}^{n}r_{i}a_{ij}(y_{R,j}+y_{U,j}+y_{G,j}+y_{F,j}+y_{I,j}+y_{E,j}+y_{P,j})\\ \;+\sum_{i=1}^{n}\sum_{k=1}^{n}\sum_{j=1}^{n}r_{i}a_{ik}a_{kj}(y_{R,j}+y_{U,j}+y_{G,j}+y_{F,j}+y_{I,j}+y_{E,j}+y_{P,j})\\ \;+\cdots \end{array}$$ (14)

In Formula (14), subscripts i, j and k represent the production sector; r_i_ represents the element of R. a_ij_, a_ik_, a_kj_… are all elements in A. The final demand Y includes rural household consumption(Y_R_), urban household consumption(Y_U_), government consumption(Y_G_), fixed capital formation(Y_F_), increase in inventory(Y_I_), export(Y_E_), and flow to other provinces(Y_P_). y_R,i_(y_R,j_) represents the element in Y_R_, y_U,i_(y_U,j_) represents the element in Y_U_, y_G,i_(y_G,j_) represents the element in Y_G_, y_F,i_(y_F,j_) represents the element in Y_F_, y_I,i_(y_I,j_) represents the element in Y_I_, y_E,i_(y_E,j_) represents the element in Y_E_, y_P,i_(y_P,j_) represents the element in Y_P_. Formula (14) consists of multiple parts, taking r_i_a_ij_y_R,j_ as an example. r_i_a_ij_y_R,j_ represents the water consumption along the “i→j→rural household consumption” pathway. The same applies to other parts. The water-saving amount for a specific pathway is obtained by subtracting the water consumption calculated using end-year data from the water consumption calculated using beginning-year data. By sorting the water-saving amount of these production chains, we can find the key paths with the greatest impact on water saving. In the “Analysis of key water-saving paths” section of this article, SPA method is used to identify the top 30 pathways with the best water-saving effects in three provinces, displayed using a Sankey diagram.

### Structural path decomposition

Structural path decomposition (SPD) is a method generated by combining structural decomposition analysis (SDA) and structural path analysis (SPA), which can decompose the specific influence of water consumption drivers to the supply chain level, thus achieving a more microscopic analysis of water consumption changes [51]. According to Formula (13) in SPA and Formula (11) in SDA, Formula (15) can be obtained:

$$\begin{array}{l} \Delta W=(\frac{1}{2}\Delta RY_{0}+\frac{1}{2}\Delta RY_{1})+(\frac{1}{2}R_{1}\Delta Y+\frac{1}{2}R_{0}\Delta Y)\\ +(\frac{1}{2}△RA_{0}Y_{0}+\frac{1}{2}△RA_{1}Y_{1})+(\frac{1}{2}R_{1}△AY_{0}+\frac{1}{2}R_{0}△AY_{1})\\ +(\frac{1}{2}R_{1}A_{1}△Y+\frac{1}{2}R_{0}A_{0}△Y)+(\frac{1}{2}△RA_{0}A_{0}Y_{0}+\frac{1}{2}△RA_{1}A_{1}Y_{1})\\ +(\frac{1}{2}R_{1}△AA_{0}Y_{0}+\frac{1}{2}R_{0}△AA_{1}Y_{1})+(\frac{1}{2}R_{1}A_{1}△AY_{0}+\frac{1}{2}R_{0}A_{0}△AY_{1})\\ +(\frac{1}{2}R_{1}A_{1}A_{1}△Y+\frac{1}{2}R_{0}A_{0}A_{0}△Y)+\cdots \end{array}$$ (15)

In Formula (15), multiple parts are added together. The sum of the parts with *ΔR* represents the change in water consumption caused by variations in water intensity. The sum of the parts with *ΔA* represents the change in water consumption caused by variations in the direct consumption coefficient. The sum of the parts with *ΔY* represents the change in water consumption caused by variations in final demand. In the “Analysis of key water-saving paths” section of this article, SPD is used to quantify the effects of driving factors on key water-saving pathways. Y can also be expressed using Eq (7), so the *ΔW* in Formula (15) can be further decomposed. Further decomposition of Formula (15) allows for the identification of the changes in water consumption caused by variations in the structure of final demand, variations in the type of final demand, and variations in the scale of final demand. Since further decomposition of the formula would result in excessive length, it is not included here. In the “Analysis of water consumption during the intermediate production stages” section of this article, SPD is used to quantify the effects of driving factors on water consumption in each stage, including the effects of final demand structure, the final demand type, and final demand scale, and so on.

### Research object and data source

**Fig 1:**
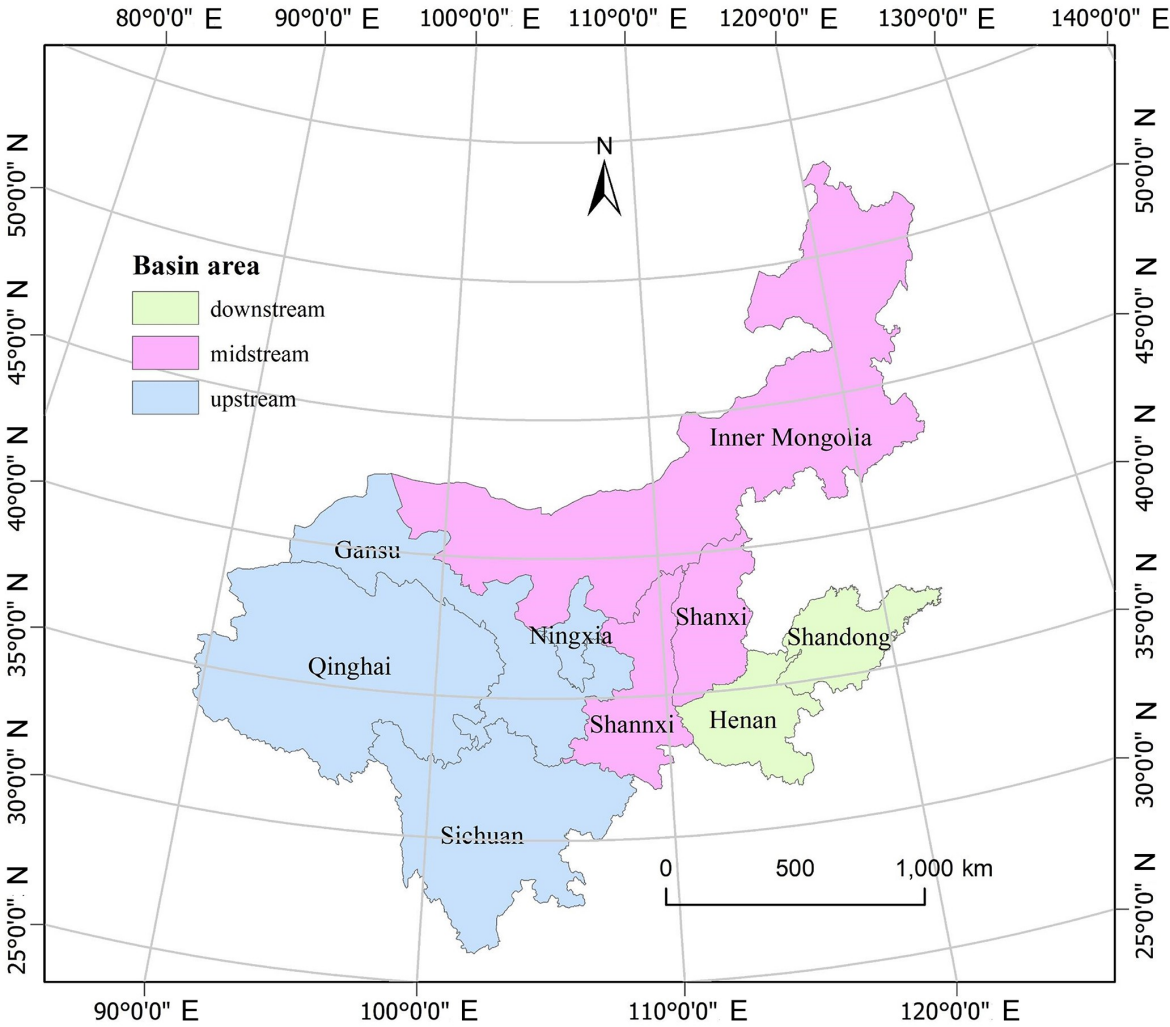
Map of the study area. Note: The map is produced based on the public domain map dataset downloaded from Natural Earth (https://www.naturalearthdata.com/). There is no modifications to the base map. The map shows the nine provinces through which the Yellow River flows. Among them, Ningxia, Gansu, Qinghai, and Sichuan provinces are in the upper reaches, Shanxi, Inner Mongolia, and Shaanxi in the middle reaches, and Henan and Shandong in the lower reaches. In this study, Ningxia, Gansu, and Henan are selected as the research objects.

This article selects typical water-deficient provinces in the Yellow River Basin, including Ningxia, Shanxi, and Henan provinces, as the research objects. The geographic location of the study area is shown in Fig 1. The methodology section explains the parts to be solved in the following text. In order to achieve the goals, the variables W, x_ij_, X, Y, Y_R_, Y_U_, Y_G_, Y_F_, Y_I_, Y_E_ and Y_P_ require enter data, while other variables can be derived based on the formulas provided in the methodology section. The data for x_ij_, X, Y, Y_R_, Y_U_, Y_G_, Y_F_, Y_I_, Y_E_ and Y_P_ come from arrays of intermediate demand, total output, final demand, rural household consumption, urban household consumption, government consumption, fixed capital formation, increase in inventory, export and flow to other provinces which are in the input-output tables. The original input-output table of each province comes from China’s regional input-output tables compiled by the National Economic Accounting Department of the National Bureau of Statistics. The beginning-year and the end-year are 2012 and 2017. Due to the classification standard differences in the two input-output tables of 2012 and 2017, this paper unifies the calculation caliber and merges the sectors of the national economy into 41 sectors. In order to eliminate the influence of price factors on input-output data in different years, it is necessary to adjust the input-output table in 2017 to the input-output table with comparable price in 2012. Adjusted input-output tables were used in this study, and the large-scale input-output tables for Ningxia, Shanxi, and Henan provinces in 2012 and 2017 can be found in the S1 Table. The data for W is obtained from the water resources bulletins for 2012 and 2017. The water consumption of sector 1 (agriculture, forestry, animal husbandry and fishery products and services) and sector 27 (construction) can directly use the relevant data in the bulletin. The water consumption of sector 2-sector 26 (industrial sector) and sector 28-sector 41 (service sector) are obtained according to the investment ratio of sector 26 (water production and supply) to each industrial sector (service sector). The water consumption of the 41 industries in the three provinces in 2012 and 2017 and the total water consumption, are presented in Table 2.

**Table 2:** Water consumption of various industries and total water consumption in Ningxia, Shanxi and Henan in 2012 and 2017 (unit: 100 million cubic meters).

Code	Sector	2012			2017		
		Ningxia	Shanxi	Henan	Ningxia	Shanxi	Henan
1	Agriculture, forestry, animal husbandry and fishery products and services	95.12	42.74	130.13	56.37	45.55	122.84
2	Coal mining and dressing products	0.49	2.67	44.71	0.43	9.97	3.74
3	Oil and gas production products	0.01	0.01	0.06	0.00	0.01	0.04
4	Metal ore mining and processing products	0.00	0.21	0.16	0.02	0.04	1.06
5	Non metallic ore and other ore mining and processing products	0.00	0.00	0.00	0.04	0.00	0.19
6	Food and tobacco	0.43	1.14	0.60	0.23	0.13	12.86
7	Textile industry	0.05	0.04	0.01	0.10	0.00	2.13
8	Textile, clothing, shoes, hats, leather, down and its products	0.03	0.02	0.01	0.01	0.00	1.48
9	Wood processing products and furniture	0.01	0.02	0.01	0.00	0.00	1.75
10	Paper making, printing and cultural, educational and sports goods	0.06	0.08	0.05	0.14	0.01	1.58
11	Petroleum, coking products and nuclear fuel products	0.50	1.07	1.05	0.09	0.23	0.32
12	Chemical products	11.15	2.02	2.93	0.51	0.65	5.18
13	Non metallic mineral products	0.18	0.70	0.52	0.07	0.12	3.25
14	Metal smelting and calendaring products	0.59	2.91	1.01	1.75	0.22	3.53
15	Metal products	0.02	0.09	0.17	0.02	0.04	1.19
16	General equipment	0.11	0.08	0.32	0.02	0.07	1.50
17	Special equipment	0.04	0.23	2.12	0.01	0.47	1.45
18	Transportation equipment	0.01	0.14	0.12	0.01	0.03	1.50
19	Electrical machinery and equipment	0.08	0.09	0.08	0.02	0.02	1.63
20	Communication equipment, computers and other electronic equipment	0.00	0.33	0.23	0.00	0.05	2.17
21	Instruments and Apparatuses	0.01	0.01	0.28	0.00	0.06	0.29
22	Other manufactured products and waste	0.00	0.04	0.01	0.01	0.00	0.04
23	Metal products, machinery and equipment repair services	0.00	0.08	0.07	0.00	0.01	0.03
24	Production and supply of electricity and heat	1.29	0.92	1.84	0.33	0.41	3.88
25	Gas production and supply	0.08	0.03	0.12	0.01	0.03	0.13
26	Water production and supply	0.56	2.58	4.05	0.69	0.90	0.03
27	Construction	1.39	0.43	18.30	0.87	0.87	12.33
28	Wholesale and retail	0.02	0.15	0.57	0.05	0.03	1.79
29	Transportation, warehousing and postal services	0.09	0.33	1.60	0.36	0.08	1.38
30	Accommodation and catering	0.06	0.84	8.36	0.18	0.40	4.17
31	Information transmission, software and information technology services	0.01	0.01	1.65	0.04	0.08	0.48
32	Finance	0.04	0.16	0.34	0.05	0.02	4.29
33	Real estate	0.02	0.07	2.35	0.07	0.11	4.55
34	Leasing and business services	0.04	0.03	0.30	0.00	0.01	1.35
35	Scientific research and technical services	0.01	0.05	1.30	0.00	0.06	0.73
36	Water conservancy, environment and public facilities management	0.01	0.03	3.37	0.02	0.16	0.24
37	Resident services, repairs and other services	0.16	0.07	0.84	0.08	0.04	1.93
38	Education	0.07	0.23	0.15	0.52	0.01	16.09
39	Health and social work	0.04	0.08	0.68	0.02	0.03	4.79
40	Culture, sports and entertainment	0.01	0.03	8.12	0.00	0.38	0.62
41	Public management, social security and social organization	0.04	0.11	0.15	0.06	0.01	5.21
Total		112.81	60.83	238.70	63.22	61.31	233.77

## Results and discussion

### Analysis of water consumption at macro aggregate level

**Fig 2:**
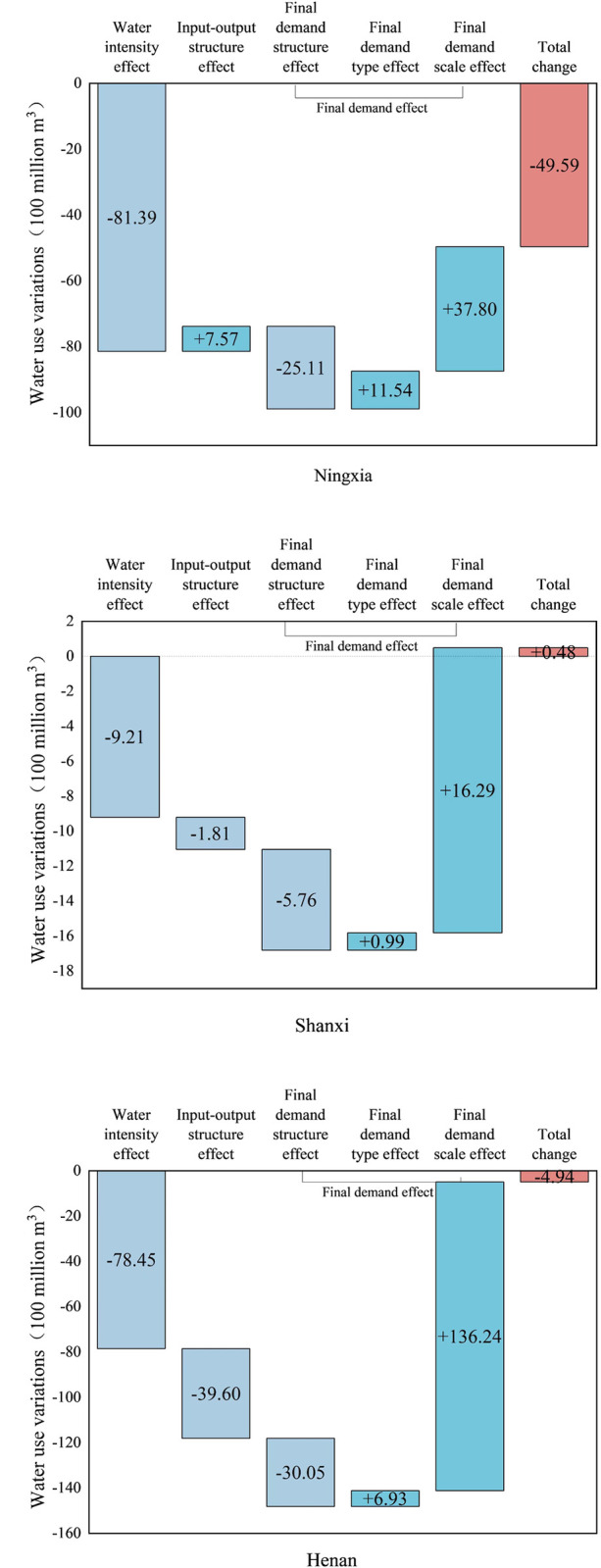
Decomposition of driving factors of total water consumption in the three provinces of the Yellow River Basin from 2012 to 2017.

The changes in water consumption in Ningxia, Shanxi and Henan from 2012 to 2017 were -49.59 million cubic meters, 0.48 million cubic meters and-494 million cubic meters, respectively, with the change ratios of -43.96%, 0.79% and -2.07%. Fig 2 shows the various influencing factors and their degree of influence on the change in water consumption in the three provinces of the Yellow River Basin. From influencing factors perspective, water intensity improved water-saving effects by 81.39%, 9.21%, and 78.45% in each province. In comparison, the final demand exerted water-saving inhibitory effects of 24.23%, 11.52%, and 113.12% in each province. These findings align with the conclusions of Zhang et al. [52], who suggested that water intensity promotes water conservation while final demand hinders it. The result of “water intensity playing a role in water conservation” indicates that the popularization of water-saving technology in the Yellow River Basin has played a positive role in water-saving work in various provinces at present, consistent with the findings of Guo et al. [53], who used input-output model to study the driving effect of virtual water in the Yellow River Basin. The main reason for the “promotion of water consumption by final demand” is primarily due to the expansion of the final demand scale and the change of the final demand type, fueled by economic growth and the technical rebound effect, resulting in higher demand for water-intensive products.

In terms of influence degree, the water saving caused by the water intensity effect in Ningxia Province is the largest, followed by Henan Province and Shanxi Province. Improving water-saving carrier evaluation standards, introducing water-saving technical regulations and implementing water intake supervision in Ningxia Province have continuously enhanced water use efficiency. Both the intensity of water use and the input-output structure in Henan province have caused water savings. This is because Henan Province pays attention to technological progress, intensive management and industrial structure optimization, all promoting water saving [54]. However, the water-saving amount caused by the effect of water intensity in Shanxi Province is the smallest, mainly caused by the small change of its own water intensity, indicating that its water-saving space is gradually shrinking and it is more difficult to reduce the water intensity.

The analysis above reveals the dynamic nature of water usage in the three provinces within the Yellow River Basin and emphasizes the various factors influencing water consumption. In terms of water extraction regulation, Ningxia province has provided proactive solutions for water conservation. The successful model in Ningxia province can serve as a reference for other regions with similar geographic and environmental conditions [55].

It is worth noting that Shanxi province exhibits relatively low water savings. Given the current limited changes in engineering technology levels, the potential for further water conservation is already minimal. To further explore water-saving potential, reforms and innovations in water management are required [56]. From a perspective of sustainable development, Shen et al. [57] propose strategies for reforming water rights systems and provide recommendations for enhancing water resource monitoring, measurement, and management systems.

Additionally, due to economic growth and the rebound effect of technology, there is an increase in demand for water-intensive products, indicating a potential dilemma in achieving sustainable water use. It may require further policy changes to regulate consumption, as suggested by some scholars [58], who propose strict water quota management as a fundamental measure to suppress unreasonable water demand. Alternatively, changing public attitudes towards water conservation could shape future water consumption patterns, as recommended by other scholars [59], who propose establishing a product water footprint labeling and certification system for key consumer products to guide consumers in choosing products with relatively lower water footprints.

This study provides a valuable analysis of the current situation while also fostering further research on how to improve water efficiency and achieve sustainable water resource management technologies and policies in these provinces, as well as in other provinces. Moreover, it is crucial to pay more attention to the potential trade-offs between economic growth and sustainable water use [60], especially in provinces like Shanxi that face greater constraints in reducing water intensity.

### Analysis of water consumption during the intermediate production stages

**Fig 3:**
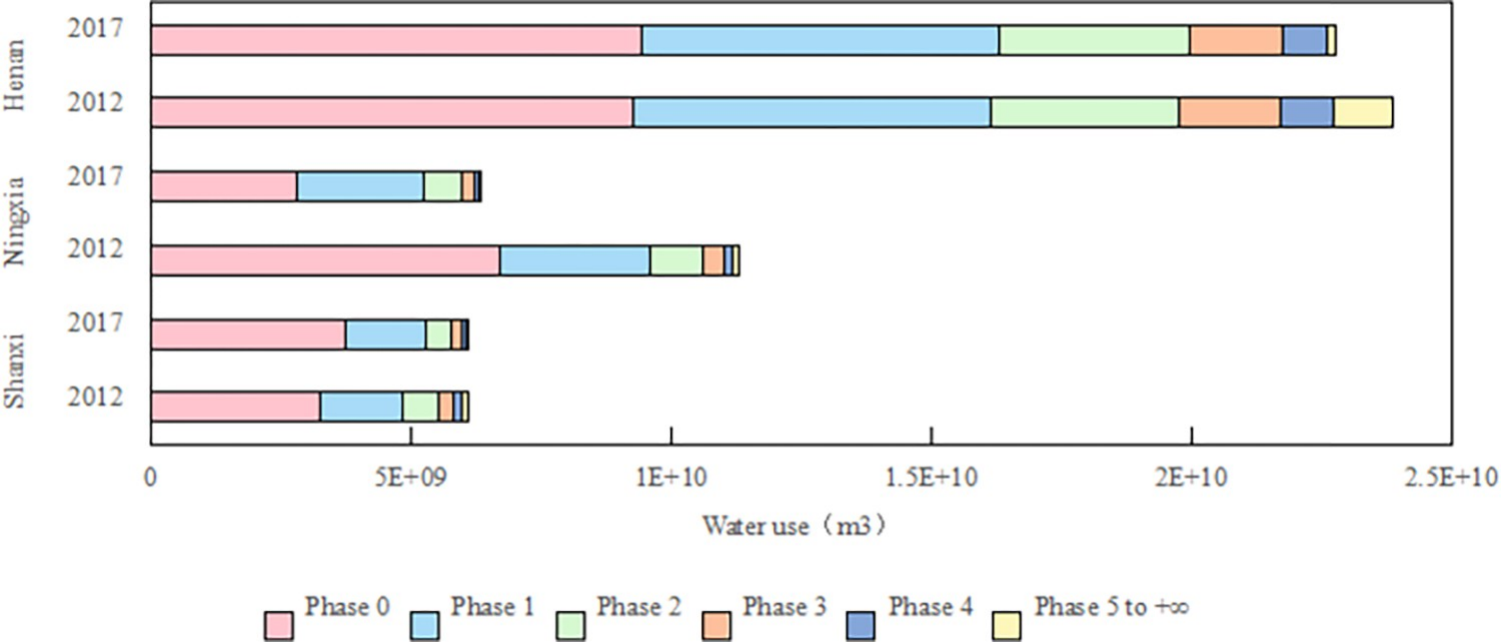
Water consumption by production stages in the three provinces in 2012 and 2017.

The water used in the 0th production stage is the direct water used by the industry to produce the final consumer goods, and the water used in the subsequent production stage is the indirect water. Fig 3 shows water consumption by production stages in the three provinces in the Yellow River Basin in 2012 and 2017. Judging from the water consumption in each production stage in 2017, the water consumption in the three provinces of the Yellow River Basin is concentrated in the 0–6 production stage, and the water consumption in the 0th production stage accounts for the largest proportion in each production stage, and then decreases step by step. Among them, Ningxia Province accounts for 44.41% in the 0th production stage and 99.27% in the 0–4 production stage. In Shanxi province, the proportion of the 0th production stage is 60.61%, and the water consumption in the 0–5 production stage is 99.44%. In Henan province, the proportion of the 0th production stage is 40.27%, and the proportion of water used in the 0–6 production stage is 99.23%.

**Fig 4:**
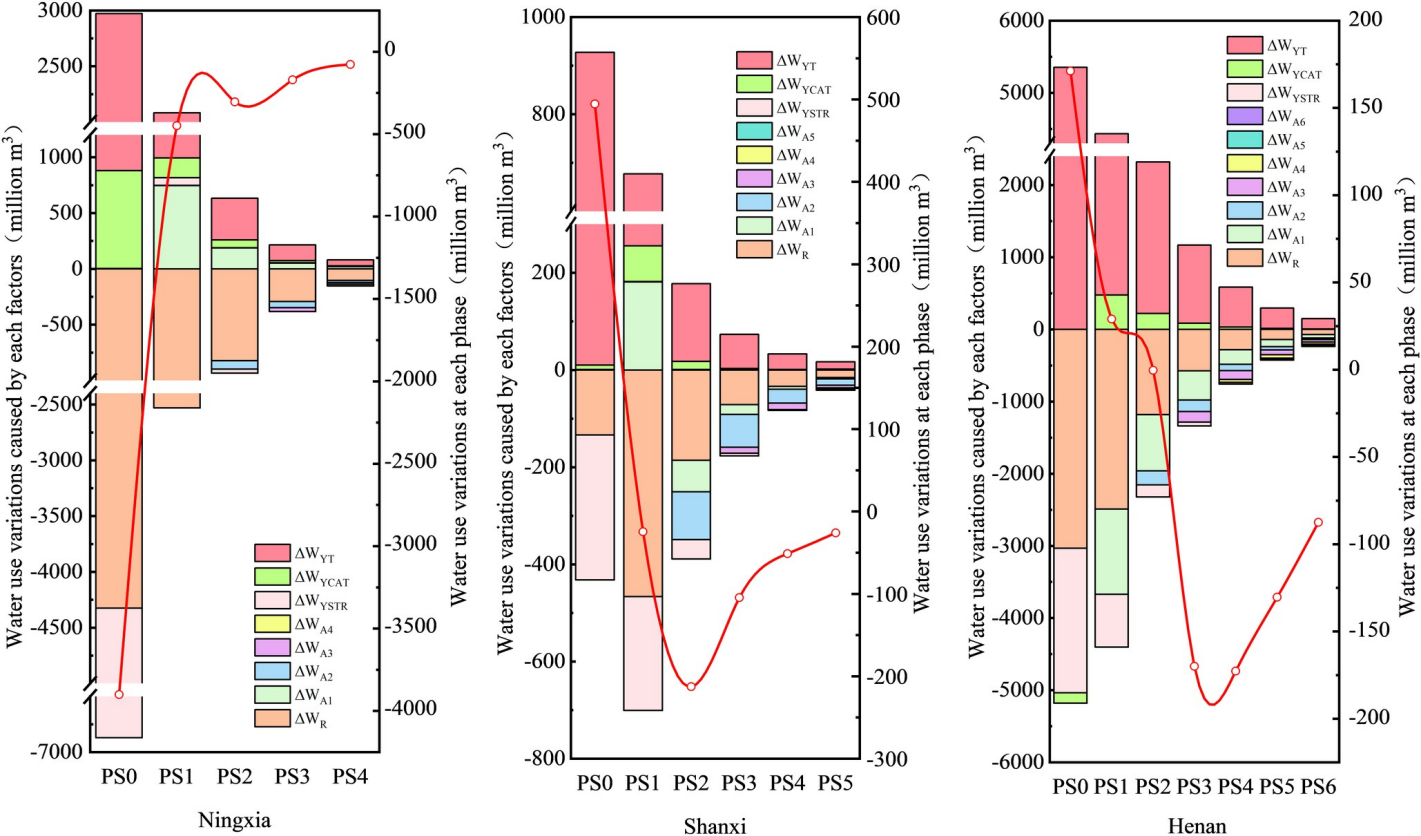
Decomposition of driving factors of water consumption at various production stages in three provinces from 2012 to 2017.

Fig 4 shows the decomposition results of the influencing factors of water consumption in the main production stages in the three provinces of the Yellow River Basin. From the perspective of influencing factors of water consumption in the main production stages, it is found that the water consumption in each production stage is mainly affected by final demand and water intensity, which aligns with the findings of Yao et al. [55]. Water intensity contributes to water saving, while final demand inhibits water saving. Among them, water consumption was reduced in the 0–4 production stages in Ningxia Province, and the change in water intensity was always the most important factor causing water saving. After the 0-stage, the water saving decreased step by step, mainly because the change of the first-order direct water consumption coefficient, final demand type and the expansion of the final demand scale jointly inhibited water saving. In the 0th production stage of Shanxi Province and Henan Province, the expansion of the final demand scale directly inhibited water saving. In contrast, in other production stages, the influence of the final demand scale was offset by the influence of the water intensity, resulting in a water-saving effect. In addition, the water consumption in the 0th and 1st production stages in Henan Province increased, and the increase of direct water consumption mainly occurred in sectors 6 (food and tobacco), 1 (agriculture, forestry, animal husbandry and fishery products and services) and 30 (accommodation and catering). For Henan Province, the focus of water saving should be placed on the sectors that lead to the overlap of water use in the 0 and 1 production stages, such as the agricultural sector [61].

These analytical findings clearly reveal the water usage patterns and key influencing factors during the intermediate production stages in the three provinces of the Yellow River Basin. They provide valuable information for the development of effective water conservation strategies and are consistent with existing research [55]. Furthermore, they highlight the need for further in-depth research in order to effectively control and optimize water usage in each production stage.

### Analysis of key water-saving paths

From the microscopic point of view, the water consumption of production chain is analyzed, and it is found that the proportion of water increase paths in the three provinces of the Yellow River Basin is greater than or equal to 50%. Among them, as a typical representative of the upper reaches of the Yellow River, 15 of the 30 paths with the largest water consumption in Ningxia Province are water-saving paths and 15 are water-increasing paths. Among the 30 paths with the largest water consumption in Shanxi Province, 12 are water-saving paths and 18 are water-increasing paths. Of the 30 paths with the largest water consumption in Henan Province, 6 are water-saving paths and 24 are water-increasing paths. Therefore, the water-saving potential of the Yellow River Basin is still great.

**Fig 5:**
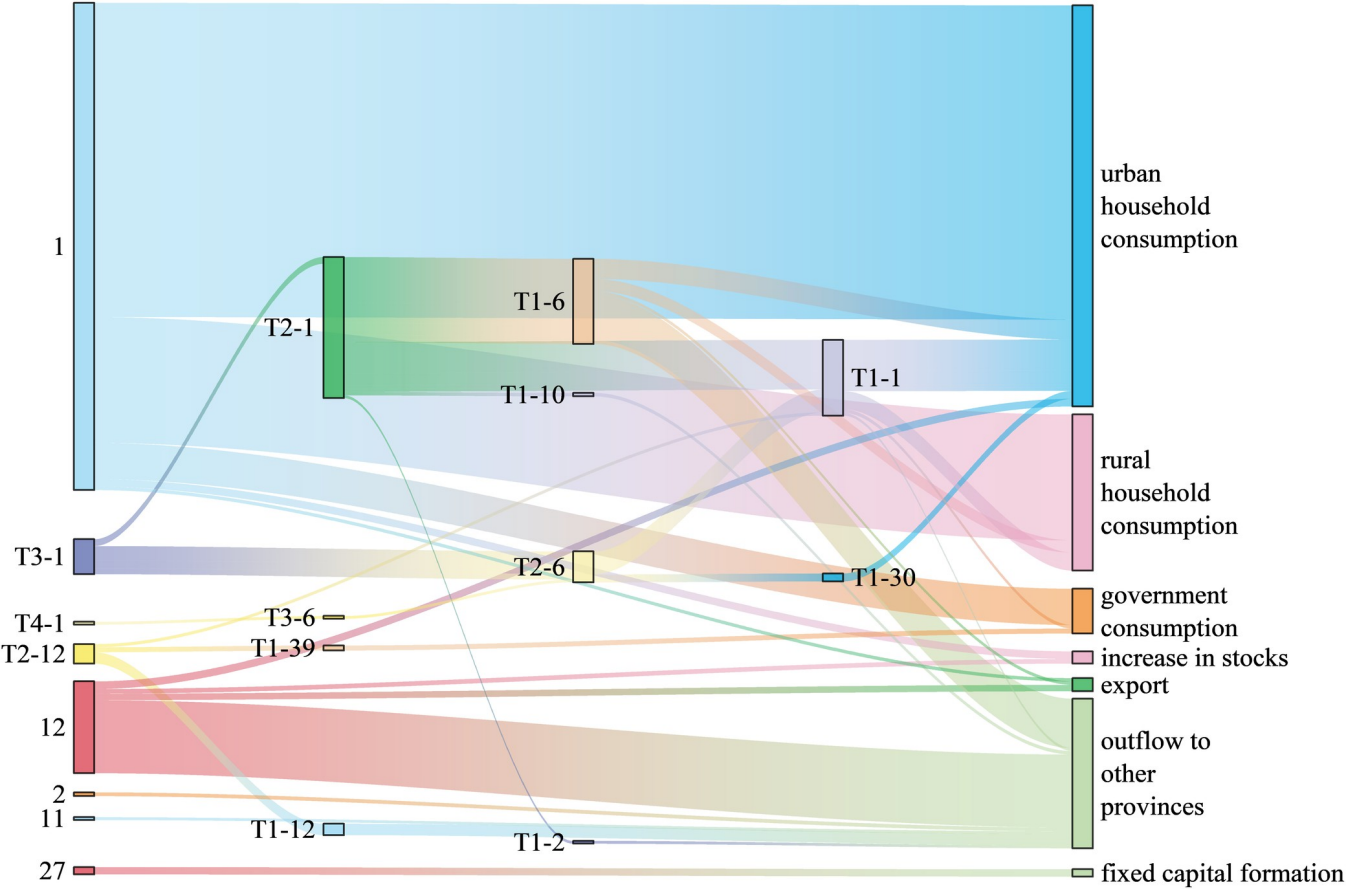
Key water-saving paths in Ningxia Province.

**Fig 6:**
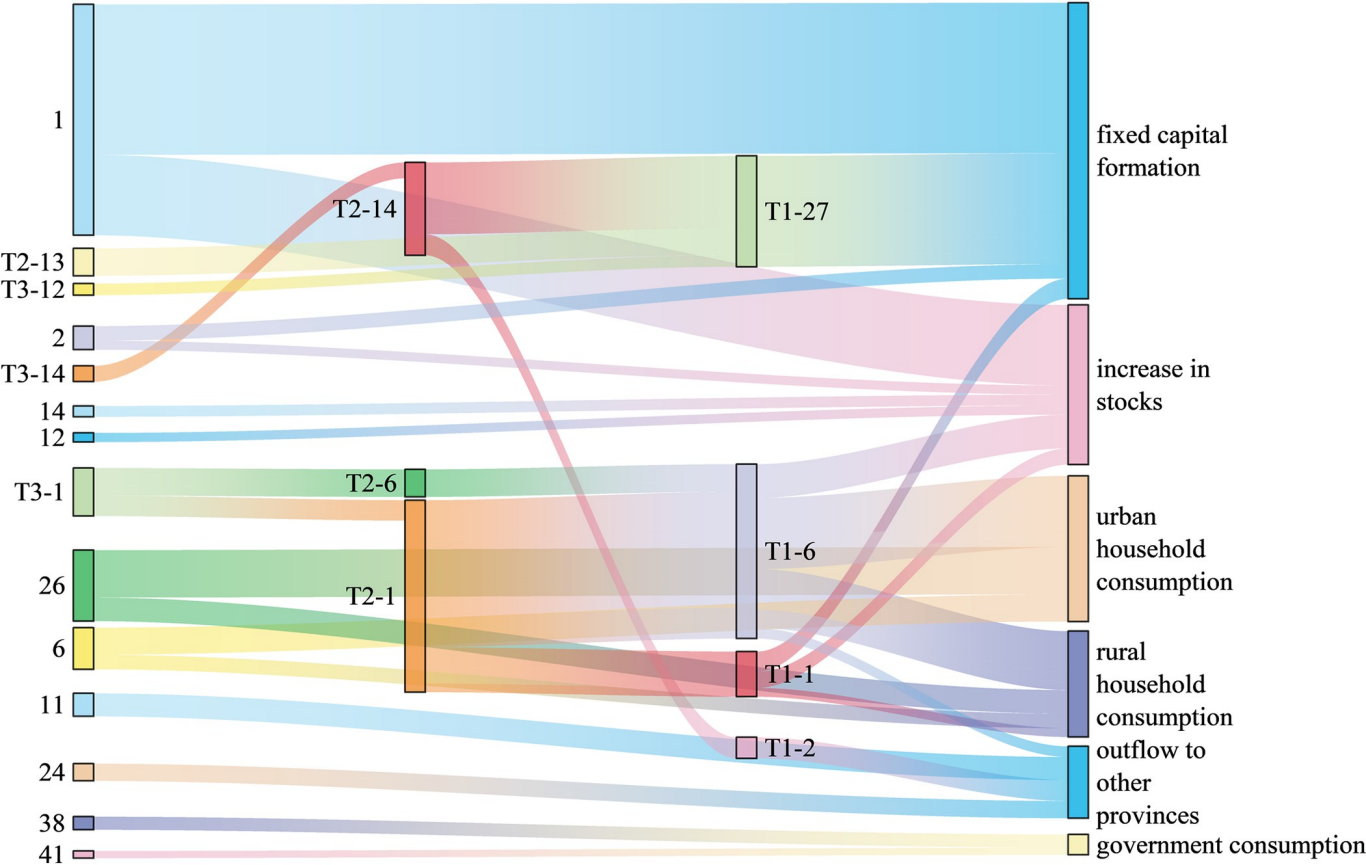
Key water-saving paths in Shanxi Province.

**Fig 7:**
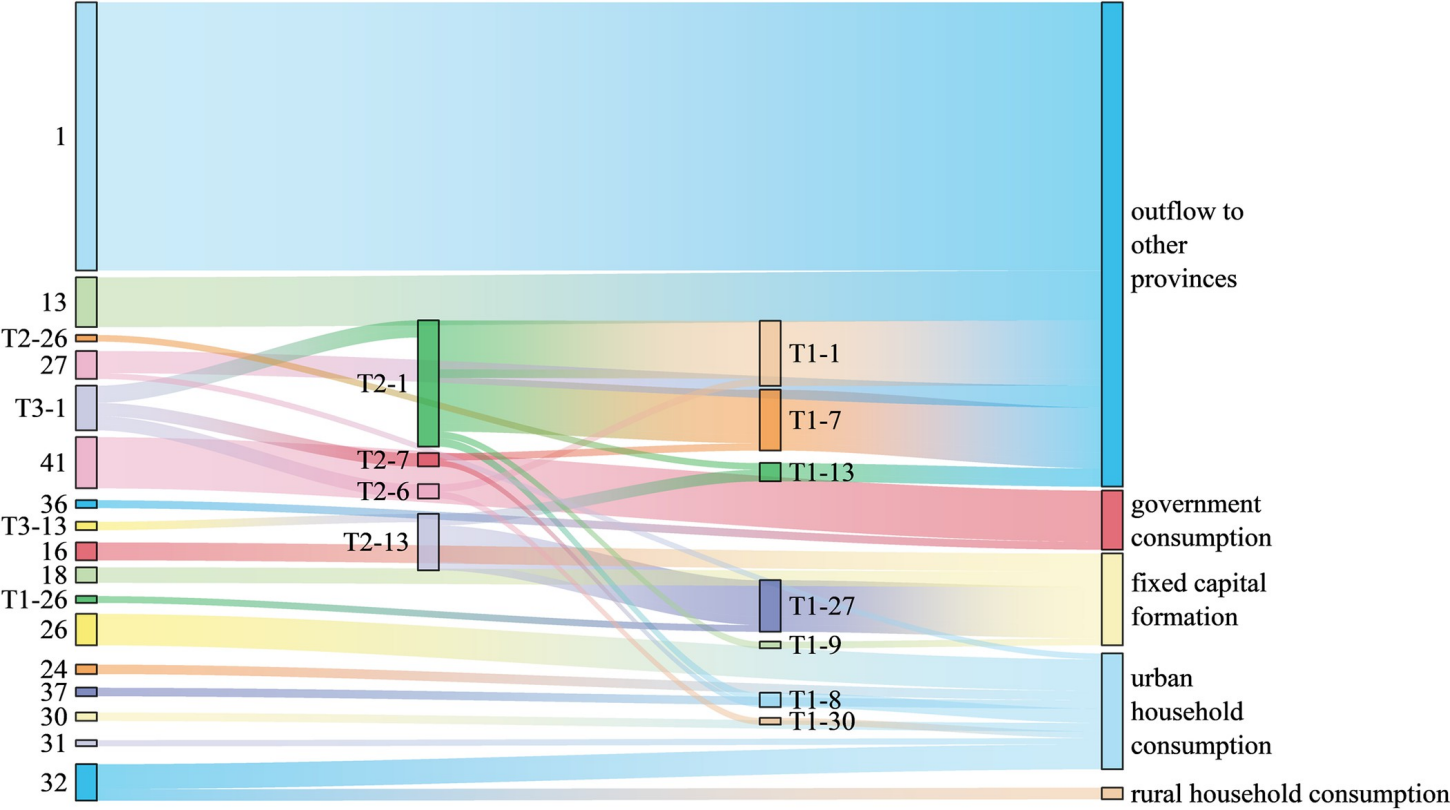
Key water-saving paths in Henan Province.

Figs 5–7 shows the 30 paths with the greatest water-saving effect in the three provinces. In terms of the length of water-saving paths, the key water-saving paths of the three provinces are all concentrated on the short production chain, and the water-saving is biased towards the demand side. Among them, there are 12 zero-order paths and 12 first-order paths in Ningxia Province. There are 14 zero-order paths and 11 first-order paths in Shanxi Province. There are 15 zero-order paths and eight first-order paths in Henan Province. In terms of consumption types, the industrial chains of rural and urban household consumption products in the three provinces account for a large proportion of water savings. In addition, in Ningxia and Henan, the industrial chains transferred out of province also save a lot of water. In Shanxi, the industrial chains where capital formation products are located save the most water. The 30 key water-saving paths are not related to export products, so it is necessary to strengthen the adjustment of export product structure. As for the sectors involved in the top 30 water-saving paths in the three provinces, the “hot” sector is sector 1 (agriculture, forestry, animal husbandry, and fishery), which is generally the starting point of key water-saving paths, consistent with the research findings of Sun et al. [62]. Of the 30 important water-saving paths in Ningxia, Shanxi and Henan provinces, 20, 15 and 11 departed from sector 1, respectively. In addition, due to the different regional characteristics, there are some differences in other hot sectors involved in the three provinces. Hot sectors in Ningxia Province also include sector 6 (food and tobacco) and sector 12 (chemical products), and there is an associated network of water resources transmission between these two sectors and sector 1, which can play a role in reducing water consumption. Most of the water-saving paths in Shanxi Province involve sectors 2 (coal mining products) and 6 (food and tobacco), which is consistent with the reality that Shanxi Province is rich in coal resources and has many coal bases. Henan Province is rich in nonmetallic mineral resources and complete in variety, so the water-saving paths related to sector 13 (nonmetallic mineral products) are the most among the three provinces. Water conservation for the mainstay industries is often the most important concern of a region’s water conservation program. Experts from China’s Ministry of Water Resources (MWR) summarized typical regional water conservation carrier construction practices in China [63] and found that local governments tend to prioritize water conservation measures for large water users, such as monitoring their water consumption data, applying differentiated water pricing that is higher than that of the general industry, and prioritizing the allocation of special financial funds to support them in the construction of water-conserving enterprises.

Table 3 is the decomposition table of the driving factors of key water-saving paths in the three provinces of the Yellow River Basin from 2012 to 2017. The following analyzes the influencing factors of key water-saving paths. On the whole, among various factors that play a water-saving role in the key water-saving paths, water intensity, direct water consumption coefficient in the first production stage and direct water consumption coefficient in the second production stage have great water-saving effects on the three provinces in the upper, middle and lower reaches of the Yellow River Basin. However, the influence of final demand varies widely. Water intensity has strong water-saving effects on key water-saving paths in the three provinces, and its contribution rates in Ningxia, Henan and Shanxi Provinces are 100.42%, 59.02% and 42.34% respectively. Final demand has a water-saving effect on key paths in Shanxi and Henan Provinces, with contribution rates of 35.06% and 28.23%, respectively. However, it suppresses water-saving efforts in Ningxia Province, reducing it by 8.64%.

**Table 3:** Decomposition of driving factors of water-saving paths in the three provinces of the Yellow River Basin from 2012 to 2017 (m^3^).

Ningxia							
ranking	ΔW	path	ΔW_R_	ΔW_A1_	ΔW_A2_	ΔW_A3_	ΔW_Y_
1	-2.11E+09	1→Urban household consumption	-1.34E+09				-7.66E+08
2	-8.45E+08	1→Rural household consumption	-5.67E+08				-2.78E+08
3	-4.91E+08	12→Flow to other provinces	-8.64E+08				3.74E+08
4	-3.06E+08	1→6→Flow to other provinces	-4.44E+08	-1.47E+08			2.84E+08
5	-2.43E+08	1→Government consumption	-1.24E+08				-1.19E+08
6	-2.07E+08	1→1→Urban household consumption	-1.47E+08	2.29E+07			-8.29E+07
7	-1.35E+08	1→6→Urban household consumption	-3.47E+08	-1.21E+08			3.33E+08
8	-9.63E+07	1→6→1→Urban household consumption	-4.23E+07	-1.00E+07	-1.86E+07		-2.53E+07
9	-8.25E+07	1→1→Rural household consumption	-6.21E+07	9.78E+06			-3.01E+07
10	-8.21E+07	1→6→Rural household consumption	-1.67E+08	-5.72E+07			1.42E+08
11	-7.63E+07	12→12→Flow to other provinces	-1.29E+08	-5.21E+06			5.78E+07
12	-5.35E+07	1→6→30→Urban household consumption	-2.62E+07	-6.57E+06	-5.68E+07		3.60E+07
13	-5.13E+07	12→Urban household consumption	-6.86E+07				1.73E+07
14	-5.09E+07	1→Increase in inventory	-1.63E+08				1.12E+08
15	-4.94E+07	27→Fixed capital formation	-1.00E+08				5.07E+07
16	-4.36E+07	12→Export	-8.83E+07				4.47E+07
17	-3.91E+07	1→6→1→Rural household consumption	-1.77E+07	-4.26E+06	-7.98E+06		-9.19E+06
18	-3.35E+07	12→39→Government consumption	-3.21E+07	1.47E+06			-2.91E+06
19	-3.12E+07	12→Increase in inventory	-3.06E+07				-6.02E+05
20	-2.61E+07	1→1→6→Flow to other provinces	-4.96E+07	8.66E+06	-1.57E+07		3.05E+07
21	-2.52E+07	2→Flow to other provinces	5.63E+06				-3.09E+07
22	-2.43E+07	1→1→Government consumption	-1.34E+07	1.98E+06			-1.29E+07
23	-2.38E+07	1→10→Flow to other provinces	-7.10E+06	-9.35E+06			-7.35E+06
24	-2.29E+07	1→Export	-3.71E+08				3.48E+08
25	-2.23E+07	1→6→Export	-1.69E+07	-4.95E+06			-5.01E+05
26	-2.13E+07	12→1→Urban household consumption	-1.87E+07	1.47E+06			-4.04E+06
27	-2.01E+07	1→1→1→Urban household consumption	-1.62E+07	2.57E+06	2.51E+06		-9.00E+06
28	-1.90E+07	1→2→Flow to other provinces	-6.73E+06	-3.25E+06			-9.00E+06
29	-1.89E+07	1→6→6→1→Flow to other provinces	-1.26E+07	-3.56E+06	-7.28E+06	-5.08E+06	9.60E+06
30	-1.87E+07	11→Flow to other provinces	-2.25E+07				3.85E+06
Shanxi							
ranking	ΔW	path	ΔW_R_	ΔW_A1_	ΔW_A2_	ΔW_A3_	ΔW_Y_
1	-2.05E+08	1→Fixed capital formation	-1.33E+07				-1.92E+08
2	-1.10E+08	1→Increase in inventory	-2.29E+07				-8.67E+07
3	-7.62E+07	14→27→Fixed capital formation	-7.88E+07	-1.85E+07			2.11E+07
4	-6.47E+07	26→Urban household consumption	-4.04E+07				-2.43E+07
5	-5.63E+07	1→6→Urban household consumption	-2.40E+07	-5.65E+07			2.41E+07
6	-5.54E+07	1→6→Rural household consumption	-1.15E+07	-2.66E+07			-1.74E+07
7	-4.61E+07	1→6→Increase in inventory	-1.84E+06	-3.79E+06			-4.04E+07
8	-3.73E+07	13→27→Fixed capital formation	-3.73E+07	-1.19E+07			1.18E+07
9	-3.69E+07	6→Urban household consumption	-3.89E+07				2.08E+06
10	-3.21E+07	26→Rural household consumption	-1.90E+07				-1.32E+07
11	-3.13E+07	11→Flow to other provinces	-3.79E+07				6.52E+06
12	-2.87E+07	14→2→Flow to other provinces	-2.84E+07	-7.49E+06			7.19E+06
13	-2.76E+07	1→1→Fixed capital formation	-1.53E+06	-5.91E+06			-2.02E+07
14	-2.37E+07	1→6→6→Urban household consumption	-2.09E+06	-4.68E+06	-1.91E+07		2.16E+06
15	-2.34E+07	24→Flow to other provinces	-1.18E+07				-1.16E+07
16	-2.19E+07	1→1→Increase in inventory	-2.46E+06	-1.04E+07			-9.11E+06
17	-2.15E+07	14→14→27→Fixed capital formation	-1.86E+07	-3.64E+06	-5.01E+06		5.71E+06
18	-1.99E+07	6→Rural household consumption	-1.84E+07				-1.50E+06
19	-1.97E+07	20→Fixed capital formation	-1.79E+07				-1.77E+06
20	-1.78E+07	38→Government consumption	-1.82E+07				3.20E+05
21	-1.68E+07	1→1→6→Urban household consumption	-2.53E+06	-1.09E+07	-5.98E+06		2.58E+06
22	-1.59E+07	12→27→Fixed capital formation	-1.50E+07	-7.62E+06			6.77E+06
23	-1.47E+07	14→Increase in inventory	-9.65E+06				-5.05E+06
24	-1.44E+07	1→6→Flow to other provinces	-1.71E+06	-3.88E+06			-8.84E+06
25	-1.38E+07	1→6→6→Rural household consumption	-1.03E+06	-2.29E+06	-8.92E+06		-1.56E+06
26	-1.26E+07	12→Increase in inventory	-4.58E+06				-7.99E+06
27	-1.25E+07	2→Increase in inventory	3.70E+07				-4.94E+07
28	-1.20E+07	1→1→Rural household consumption	-5.67E+06	-2.54E+07			1.91E+07
29	-1.10E+07	1→1→6→Rural household consumption	-1.23E+06	-5.18E+06	-2.77E+06		-1.86E+06
30	-9.82E+06	41→Government consumption	-1.14E+07				1.60E+06
Henan							
ranking	ΔW	path	ΔW_R_	ΔW_A1_	ΔW_A2_	ΔW_A3_	ΔW_Y_
1	-1.62E+09	1→Flow to other provinces	-1.89E+08				-1.43E+09
2	-3.10E+08	41→Government consumption	-3.44E+08				3.48E+07
3	-3.01E+08	13→Flow to other provinces	-5.95E+08				2.94E+08
4	-2.96E+08	1→1→Flow to other provinces	-3.03E+07	-5.82E+07			-2.08E+08
5	-2.72E+08	1→7→Flow to other provinces	-3.63E+07	-1.25E+08			-1.11E+08
6	-2.20E+08	13→27→Fixed capital formation	-2.34E+08	-1.28E+08			1.42E+08
7	-1.90E+08	26→Urban household consumption	-1.37E+08				-5.32E+07
8	-1.51E+08	32→Urban household consumption	-1.82E+08				3.11E+07
9	-1.33E+08	27→Flow to other provinces	-4.18E+07				-9.17E+07
10	-1.08E+08	16→Fixed capital formation	-6.81E+07				-4.04E+07
11	-9.39E+07	18→Fixed capital formation	-4.90E+07				-4.49E+07
12	-7.09E+07	13→13→Flow to other provinces	-1.29E+08	-8.22E+06			6.59E+07
13	-7.06E+07	32→Rural household consumption	-5.73E+07				-1.32E+07
14	-5.75E+07	24→Urban household consumption	-4.34E+07				-1.42E+07
15	-5.27E+07	1→1→1→Flow to other provinces	-4.94E+06	-9.23E+06	-7.37E+06		-3.12E+07
16	-5.16E+07	37→Urban household consumption	-1.56E+08				1.04E+08
17	-5.12E+07	1→1→7→Flow to other provinces	-5.73E+06	-1.13E+07	-1.72E+07		-1.70E+07
18	-5.11E+07	30→Urban household consumption	-7.45E+07				2.34E+07
19	-5.06E+07	13→13→27→Fixed capital formation	-5.12E+07	-2.69E+06	-2.88E+07		3.20E+07
20	-5.04E+07	1→8→Urban household consumption	-8.41E+06	-5.24E+07			1.04E+07
21	-4.82E+07	36→Government consumption	-1.21E+08				7.32E+07
22	-4.55E+07	1→6→1→Flow to other provinces	-4.57E+06	-4.90E+06	-5.12E+06		-3.09E+07
23	-4.44E+07	1→7→7→Flow to other provinces	-9.12E+06	-3.50E+07	2.89E+07		-2.91E+07
24	-4.16E+07	1→9→Fixed capital formation	-1.23E+07	9.54E+06			-3.88E+07
25	-4.11E+07	26→27→Fixed capital formation	-4.86E+07	-1.23E+07			1.97E+07
26	-4.08E+07	1→6→30→Urban household consumption	-1.24E+07	-1.49E+07	-3.01E+07		1.65E+07
27	-4.00E+07	26→13→Flow to other provinces	-2.34E+07	-3.76E+07			2.11E+07
28	-3.76E+07	31→Urban household consumption	-3.70E+07				-6.58E+05
29	-3.59E+07	1→7→8→Urban household consumption	-9.07E+06	-3.60E+07	-1.65E+06		1.09E+07
30	-3.56E+07	27→Urban household consumption	-8.92E+06				-2.66E+07

As a typical representative of the upstream region, water intensity in sectors supplying primary products in Ningxia Province exerts a water-saving effect. Specifically, the variations in water intensity of sector 1 (agriculture, forestry, animal husbandry, and fishery products and services), sector 2 (coal mining products), sector 11 (petroleum, coke products, and nuclear fuel processing), and sector 27 (construction) play a vital role in promoting water-saving in Ningxia Province. On the other hand, the changes in final demand for sector 6 (food and tobacco), sector 7 (textile products), sector 11 (petroleum, coke products, and nuclear fuel processing), sector 12 (chemical products), and sector 30 (accommodation and catering) are the primary factors inhibiting water-saving in Ningxia Province. The water-saving effect of final demand is greater than that of water intensity for the following pathways: 2→flow to other provinces, 1→10→flow to other provinces and 1→2→flow to other provinces. This indicates a decrease in out-of-province demand for the products provided by sector 2 (coal mining products) in Ningxia province, which can be attributed to the Ningxia Province’s efforts to locally transform coal resources and directly sell electricity as a substitute for coal, thereby promoting the transformation of the coal industry.

As a typical representative of the middle reaches of the Yellow River, the water-saving effect of water intensity of Route 14, Route 21 and Route 28 in Shanxi Province cannot offset the effect of increasing water demand, which shows that Shanxi Province needs to rely on the change of direct consumption coefficient to support the water-saving of industrial chains, so it needs to rely on the reorganization of industrial investment ratio to achieve the purpose of water-saving. In addition, the water intensity of sector 2 (coal mining and dressing products) in the key water-saving paths of Shanxi Province still plays a role in increasing water consumption, and the work of saving water and increasing efficiency in the coal industry in Shanxi Province has a long way to go.

In Henan province, the water intensity effect in the raw material sector, i.e., the agricultural sector of Routes 20, 26, and 29, cannot fully offset the effect of increased water use due to an increase in the size of final demand. Therefore, Henan province should intensify its efforts in agricultural water conservation.

To summarize the results of this section, there is still significant water-saving potential in the Yellow River Basin, and factors such as final demand and water intensity play crucial roles in water-saving paths. For example, in certain water-saving paths in Ningxia province, the water-saving effect of final demand surpasses that of water intensity. This is attributed to Ningxia province’s efforts in locally transforming coal resources and directly selling electricity as a substitute for coal, which promotes water conservation initiatives. In the case of Shanxi province, the water-saving effects of water intensity in some paths are unable to counterbalance the increase in water demand resulting from expanding final demand. This indicates that Shanxi province may need to rely on changes in the direct consumption coefficient to support water-saving in the industrial chains by reorganizing the proportion of industrial investment. In Henan province, the water intensity effects of certain paths in the agricultural sectors are also insufficient to fully offset the increase in water demand brought about by the expansion of the final demand. This emphasizes the need for Henan province to intensify efforts in agricultural water conservation, such as improving water-saving irrigation techniques and implementing agricultural water pricing reforms [64]. Therefore, for these three provinces, a crucial task from a micro water-saving perspective is to deeply analyze key factors such as final demand and water intensity and adjust relevant decisions and measures accordingly. In line with the research conducted by Du et al. [65], adjusting the imbalanced provincial consumption structure and meeting the direct production demand of downstream provinces will contribute to reducing water consumption in China. The water-saving effect of water intensity is considered a technological effect. Technology has always been a factor in restraining the growth of water consumption. Provinces with lower technological effects should learn from those with higher technological effects, such as studying the case of Sichuan (another province in the basin) and enhancing technological collaboration to decouple water consumption and economic growth [66]. Improving water-saving facilities, such as drip irrigation equipment and rainwater collection systems, is crucial [67].

## Conclusions

The water driving factors and key water-saving paths in typical provinces of the Yellow River Basin are explored in this study. It reveals that water intensity and final demand are the dominant factors, with indirect water consumption accounting for a significant proportion of the total water usage, with indirect water consumption exceeding 50% in 2017. The key water-saving paths are generally shorter and centered around the agricultural sector, with the water-saving effects varying among specific sectors and industrial chains based on regional differences. These findings suggest the need to encourage technological innovation and implement agricultural water management to develop region-specific strategies for water-saving along the industrial chains [68]. By reducing the water consumption throughout the product life cycle and promoting the integration of water systems along the industrial chains, the overall water utilization efficiency can be improved.

## Supporting information

S1 Table Original input-output tables of Ningxia, Shanxi and Henan in 2012 and 2017. (XLSX)
